# Targeting CD36 as Biomarker for Metastasis Prognostic: How Far from Translation into Clinical Practice?

**DOI:** 10.1155/2018/7801202

**Published:** 2018-07-04

**Authors:** Ana-Maria Enciu, Eugen Radu, Ionela Daniela Popescu, Mihail Eugen Hinescu, Laura Cristina Ceafalan

**Affiliations:** ^1^Victor Babes National Institute of Pathology, 99-101 Splaiul Independentei, Sector 5, 050096 Bucharest, Romania; ^2^Carol Davila University of Medicine and Pharmacy, No. 8 B-dul Eroilor Sanitari, Sector 5, 050474, Bucharest, Romania

## Abstract

Metastasis requires cellular changes related to cell-to-cell and cell-to-matrix adhesion, immune surveillance, activation of growth and survival signalling pathways, and epigenetic modifications. In addition to tumour cells, tumour stroma is also modified in relationship to the primary tumour as well as to distant metastatic sites (forming a metastatic niche). A common denominator of most stromal partners in tumour progression is CD36, a scavenger receptor for fatty acid uptake that modulates cell-to-extracellular matrix attachment, stromal cell fate (for adipocytes, endothelial cells), TGF*β* activation, and immune signalling. CD36 has been repeatedly proposed as a prognostic marker in various cancers, mostly of epithelial origin (breast, prostate, ovary, and colon) and also for hepatic carcinoma and gliomas. Data gathered in preclinical models of various cancers have shown that blocking CD36 might prove beneficial in stopping metastasis spread. However, targeting the receptor in clinical trials with thrombospondin mimetic peptides has proven ineffective, and monoclonal antibodies are not yet available for patient use. This review presents data to support CD36 as a potential prognostic biomarker in cancer, its current stage towards achieving* bona fide *biomarker status, and knowledge gaps that must be filled before further advancement towards clinical practice.

## 1. Introduction

Metastasis is a rather inefficient process if the number of circulating tumour cells is to be compared with the number of clinically overt metastatic sites [1]. From vascular invasion to secondary site tumour initiation, a cell can go through changes in cell-to-cell and cell-to- matrix adhesion profile, face immune surveillance systems, activate growth and survival signalling pathways, and undergo epigenetic modifications. To be effective, these changes must occur in a time-dependent manner, modifying the cell phenotype for survival in new microenvironments. Under these conditions, how much of the primary tumour is recapitulated in a metastasis? And how can we predict whether or when a primary tumour would seed secondary sites?

Perhaps surprisingly, scrutinizing the less investigated stromal tumour tissue—or the modern and reinvented “stromal metastatic niche”—could provide some answers. The metastatic niche has been defined as “extracellular matrix, nonmalignant cells, and the signalling molecules they produce” [2]. Different in composition and less characterized than its counterpart—the primary tumour niche—the stromal metastatic niche recently underwent a shift in perspective to a “premetastatic niche,” prepared in advance by conditioned infiltrating monocytes. This premetastatic niche, as yet unoccupied by tumour cells, is thought to create a tumour friendly environment to enhance the survival chances of invading cells [3].

A common denominator of most stromal partners playing a role in tumour progression is expression of CD36—a scavenger receptor for fatty acid (FA) uptake that modulates cell-to- extracellular matrix attachment, stromal cell fate (for adipocytes, endothelial cells), TGF*β* activation, and immune signalling [4]. Unlike its well-known and better-studied binding partners (thrombospondins (TSPs) 1 and 2) but with controversial involvement in cancer progression, CD36 is increasingly emerging as a prognostic marker associated with the metastatic process. Even more so, its presence seems not to be limited to tumour stroma, as the number of reports describing it on tumour cells is increasing.

Several gaps remain in CD36-related knowledge. Learning from paths travelled in understanding other receptors as cancer-related biomarkers, one can argue that there are still plenty of blank spots on the CD36 cancer-related map. In cancer studies, CD36 is investigated mostly in relationship with TSPs, a family of matrix proteins acting as ligands, and most data are related to TSP interactions and, to a lesser extent, TGFß. Only a handful of papers have been published directly addressing CD36, almost all measuring levels of expression in relationship to tumour growth and metastasis. Furthermore, lessons from myeloproliferative diseases and gliomas suggest that mutated receptors do not require a ligand to be active. In the case of CD36, constitutive activation, regardless of TSP expression within the tumour or tumour niche, could be largely overlooked if the main focus falls on the ligand rather than the receptor.

In addition, a parallel analysis of stromal CD36 versus tumour CD36 is usually missing from the scientific argument in most original articles. Last, but not least, studies in animal models using athymic mice remove a key player from the stroma, the immune cell, yielding an incomplete tumour–stroma interaction panel.

What is the ultimate goal in CD36 research? One size fits all? Probably not. That pattern has not yet been the case for any molecular intervention in diagnostics, prognostics, or therapy. However, the chase for a single but efficient antimetastatic molecular target is justifiable from several perspectives. First and foremost, time is gained for diagnostic and therapeutic intervention. Second, absence of metastases will translate into an absence of cellular polymorphisms derived from environmental change. Thus, molecular therapies addressing the primary tumour could have a greater impact on relapse and survival rates.

How far is CD36 from validated status as a biomarker and what type of biomarker would best fit are the questions the present review will attempt to answer, after analysing the evidence tying CD36 to the metastatic process and the translation into knowledge towards clinical practice.

## 2. CD36 Distribution and Functions in Normal Tissues

CD36, also known as platelet glycoprotein (GP) 4/, FA translocase (FAT)/, scavenger receptor class B member 3 (SCARB3)/, GP88, GPIIIB, or GPIV [5], is an integral membrane GP encoded by the CD36 gene and belonging to the scavenger receptor family. The intracellular trafficking of the molecule requires different degrees of glycosylation, with the heavy glycosylated protein being exposed on the cell membrane [6, 7]. When exposed, CD36 may associate with other transmembrane proteins, such as integrins (*β*1, *β*2, and *β*5) and tetraspanins (CD9, CD81) [8]. Its intracellular domains associate with members of the src family of tyrosine kinases, such as fyn, lyn, and, yes, a molecular interaction most probably mediated by lipids in the context of lipid rafts [9]. Supramolecular assembly of CD36 into nanoclusters at the plasma membrane, even in the absence of ligands, is important for downstream fyn signalling [10]. The extracellular domain binds to a vast array of ligands, which accounts for the diversity of signal transduction outcomes: (i) adhesive glycoproteins of the TSP family [11]; (ii) collagenic proteins (collagens I and IV) [5]; (iii) lipid ligands (anionic or oxidized phospholypids –PL), native and oxidized lipoproteins [8], FAs); and (iv) peptides such as hexarelin or fibrillar A*β* amyloid species [12].

As a surface protein, CD36 is widely distributed. Found on platelets and monocytes/macrophages, it is involved in cellular activation. It not only mediates the initial binding of platelets on extracellular matrix protein like collagen I [5] and TSP-1 but also triggers signal transduction, inducing an oxidative burst in monocytes [13]. In addition, it is present on erythrocytes, where it mediates adherence of* Plasmodium falciparum*–infected erythrocytes.

CD36 expression was also detected in differentiated adipocytes [14].* In vitro *studies proved that CD36 is located in lipid rafts, along with caveolin-1, mediating FA uptake [15]. Both functional studies with CD36 cross-linking agents and disruption of lipid rafts stop the transport of long-chain FAs [14].

In skeletal muscle, CD36 expression on the cell surface is an important mechanism for FA uptake and short-term regulation through subcellular redistribution [16]. However, CD36 is also found in the mitochondria, where it is responsible for FA oxidation [17]. CD36 expression is regulated by both insulin and contraction, which promotes the translocation of intracellular stored CD36 to the plasma membrane. Increased expression can contribute to lipid accumulation in heart and skeletal muscle [18].

CD36 also has been described on endothelial cells of human dermis, but only in the microvasculature and not in the large vessels [19] and in caveolin-rich membranes isolated from lung endothelium [20]. It is also present on normal mammary epithelial cells [21], which prompted its investigation in breast cancer, as discussed below.

Another type of epithelial cell expressing CD36 is the taste receptor cell within lingual taste buds in tongue of rodents [22], pigs, and humans [23]. CD36 expression is restricted to only the lingual papillae where it has been localized at the apical side of the circumvallate [22] and foliate taste buds [23]. Its expression is lipid-mediated, changing the attraction for fat during a meal [24]. Only lipid discrimination is affected in CD36-null mice [22].

CD36 is expressed in the brush border membrane of duodenal and jejunal enterocytes [25] in both mice and humans [26]. Moreover, early after lipid ingestion, CD36 disappears from the luminal side of intestinal villi [27]. Similar to adipocytes, CD36 is located in lipid rafts, where it colocalizes with caveolin-1.* In vitro *studies showed that caveolin-1 is required for the transport of CD36 to the apical membrane, thus regulating its surface availability for FA uptake [28]. Moreover, brush border caveolae provide the absorptive surface for dietary FA. Studies performed on caveolin-1 knockout mice proved that FA absorption was compromised and the animals could not gain weight [25].

Also at the intestinal level, CD36 was detected on enteroendocrine cells secreting secretin and cholecystokinin in the mucosa of the duodenum, jejunum, and proximal ileum on both apical membranes and cytosolic granules [29].

In mouse liver, CD36 is expressed on hepatocytes, endothelial cells, and Kupffer cells [30] and the expression is increased by starvation [8] and aging, especially when associated with a high-fat diet. Insulin increases CD36 expression in the liver [31] by activating PPAR*γ*, an upstream regulator of CD36 expression. Enhanced expression and subsequent fat uptake and triglyceride (TG) accumulation may accelerate progression of nonalcoholic fatty liver disease [32], insulin resistance, and type 2 diabetes [33].

In the normal brain, CD36 expression is low, but it is upregulated upon stroke due to monocyte-macrophage infiltration. It appears that CD36 contributes to acute ischemic brain injury during the inflammatory phase and is involved in phagocytosis during the recovery phase [7].

In ovary, CD36 is found on serous ovarian epithelial cells [34], contributing to angiogenesis and folliculogenesis [35].

## 3. Functions of CD36: Lessons Learned from the CD36 Knockout Mouse Model

CD36 was first identified as platelet GPIV, due to thrombocytes' ability to bind TSP [36]. Later, its overlapping structure with leukocyte differentiation antigen CD36 was demonstrated [37]. In the following years, its role in platelet activation [38] and cell adhesion [39] was investigated. Not long after, its involvement in translocation of long-chain FAs was reported [40].

Febbraio and collaborators have played an important role in understanding CD36 functions by creating mice with a null mutation in the CD36 gene through targeted homologous recombination [41]. The animal model was subsequently used by a large number of researchers, with 528 citations recorded to date (ISI Web of Science Core Collection, searched on March 13, 2018). Some of the significant insights provided by CD36-/- mouse experiments include regulation of CD36 by PPAR-gamma [42] inhibition of angiogenesis* in vitro *and* in vivo*; induction of apoptosis by TSP-1 via activation of CD36, p59fyn, caspase- 3–like proteases, and p38 mitogen-activated protein kinases [43]; understanding of atherosclerotic lesion development [44, 45] contribution to uptake and use long-chain FAs [46] diet response [47], serving as an advanced glycation end-products receptor [48] orosensory perception of long-chain FAs [49]. Conversely, mice were also engineered to overexpress CD36 in specific tissues by using the promoter of the muscle creatine kinase gene, resulting in enhanced FA oxidation, reduced plasma TGs and FAs, and increased plasma glucose and insulin [50].

More recent mouse experimental models include double-knockout animals for CD36 and other genes such as leptin [51], tyrosine-protein kinase Mer [52], liver-specific signal transducer and activator of transcription (STAT)5 [53], scavenger receptor-A [54], scavenger receptor class B type I [55], heart-specific lipoprotein lipase [56], apolipoprotein E [57], and liver-type FA-binding protein [58].

The CD36 molecular structure includes two transmembrane domains located near both ends of the molecule, joined by a large extracellular region [59]; the transmembrane domains continue with small intracellular tails that are palmitoylated [60] and are important in localizing CD36 within caveolae and lipid rafts [61]. The N-glycosylated extracellular region has a binding site for TSP-1 (residues 93–120) [62] and one site for competitive binding of FA and oxidized low-density lipoprotein (ox)LDL/oxidized glycerophospholipids (residues: 150–168) [63, 64] that can bind hexarelin, one of several growth hormone–releasing peptides [65], and PfEMP1 proteins of the malaria parasite [66]. Neculai et al. [67] found, through an analogy with the crystal structure of structurally related LIMP-2 that they described, a notable feature of the CD36 extracellular domain: a tunnel, mainly comprising hydrophobic residues, spanning its entire length and apparently able to selectively transfer cholesterol esters from the extracellular environment to the outer leaflet of the cell membrane. Thus, future, more detailed structural studies of CD36 could provide actionable targets for therapies for diseases involving this molecule and its numerous, highly variable binding partners.

Other studies have contributed to identifying and understanding the role of CD36 association with other membrane or intracellular molecules. An interesting example is the discovery of CD36 as a regulator of Toll-like receptors 4 and 6 heterodimer assembly that can subsequently trigger inflammatory signalling in microglia [68]. Such data suggest that CD36 can make a major contribution to sterile inflammation in response to atherogenic lipids and amyloid-beta.

Interesting avenues of research might also be opened by a few recent studies that identified a role for noncoding RNA molecules in CD36 expression regulation with functional consequences. miR-758-5p decreases lipid accumulation in foam cells via regulating CD36-mediated cholesterol uptake [69], long noncoding RNA MALAT1 regulates oxLDL-induced CD36 expression via activating *β*-catenin [70], and uc.372, an ultraconserved RNA belonging to the class of long noncoding RNAs, regulates expression of genes related to lipid synthesis and uptake, including CD36, via suppression of specific miR molecule maturation [71].

Following data gathered from these models and others not mentioned here, the involvement in lipid metabolism and cell-to-matrix adhesion has been confirmed for various cell types, and other functions that are site-specific have been identified and are presented briefly below.

### 3.1. Lipid Scavenger Receptor and Subsequent Impact on Lipid Metabolism

CD36 has long been known as a scavenger receptor able to bind oxidized LDL (oxLDL) and HDL (oxHDL) [72, 73] but also native lipoprotein molecules [74]. CD36 is involved in high-affinity FA uptake and processing and eventually lipid accumulation and metabolic dysfunction under excessive supply [8]. First found on monocytes and platelets, CD36 is also responsible for uptake of long-chain FA into muscle and adipose tissues and across the brush border [25]. Ligand binding activates phospholipase C, increases cytosolic Ca concentration, and activates chylomicron production [27]. CD36 also regulates the secretion of hepatic very LDL (VLDL), which may explain the correlation between CD36 protein expression and serum levels of VLDL lipid, particle number, and apolipoprotein B in humans [75]. CD36 deletion decreases VLDL output* in vivo *by increasing prostaglandins D2, F2, and E2 synthesis in the liver [51].

CD36 is a key factor in acute and adaptive regulation of muscle FA oxidation in response to a chronic metabolic stimulus and for the selection of skeletal muscle fuel under basal conditions, during acute exercise, or after muscle training [76]. In heart muscle, CD36 impacts adaptation of myocardial rhythm to energy deprivation [77]. During fasting, CD36 null mice have abnormal myocardial Ca^2+^ dynamics, phospholipid composition, and cAMP levels and associated conduction anomalies with a high incidence of sudden death [77]. Moreover, recent data have shown that myocardial CD36-mediated signal transduction activates FA *β*-oxidation [8].

*Pathology Impact*. Following the interaction of oxLDL with CD36 on intimal transmigrated macrophages, oxLDLs are internalized. They bind to the nuclear hormone receptor PPAR*ɣ* followed by the upregulation of CD36, which amplifies oxLDL uptake and foam cell formation [78]. Moreover, by stimulation of cytokine production, intima is further infiltrated and atherosclerotic lesion is formed [8].

### 3.2. Cell-to-Matrix Attachment

CD36 can bind to extracellular matrix proteins, such as collagen [5] and thrombospondin 2 in platelets and various cell lines. It also binds to TSP-1, but at concentrations higher than physiological, possibly reached in overdeveloped cancerous stromal tissue. CD47 is required for CD36 activation under TSP-1 ligation [79]. In endothelial cells, TSP binding triggers apoptosis, a mechanism bypassed in cancer, where it favours angiogenesis and tumour growth, as discussed later.

## 4. CD36 as an Early Biomarker for Metastatic Cancer

The term “biomarker” came into frequent use from the 1970s [80] and is currently defined as a “characteristic that is measured as an indicator of normal biological processes, pathogenic processes, or responses to an exposure or intervention, including therapeutic interventions” [81]. A candidate biomarker should provide measurable features, accuracy in indication for a physiologic or pathogenic mechanism, or pharmacological response to a therapeutic approach. According to the US Food and Drug Administration, an ideal biomarker should be highly sensitive and specific for a certain disease, safe, and easily measured in any biological sample, cost-effective, and able to yield accurate results [82].

Correlated with invasion of tumours and metastasis, CD36 has been repeatedly proposed as a prognostic marker in various types of cancers, mostly of epithelial origin. In the following section, we discuss the results that build up the case for CD36 as an “early prognostic marker in metastasis” and review its progress towards clinical validation.

### 4.1. Preclinical Studies

To be considered as a potential biomarker of metastatic cancer, CD36 must respect the first rule of biomarkers—to exhibit a constant change in disease versus health, in this case, in metastatic cancer versus normal paired control tissue. Indeed, the expression of CD36 has been demonstrated to be* increased *in human tumour cell lines, human biopsies from ovarian tumours [83], gastric cancer [84], glioblastoma [85], and oral squamous carcinoma [86]. In contrast, consistent with data from tumour growth mechanism studies, CD36 has been reported to be* decreased *in endothelial cells, as a bypass program of its antiangiogenic effect [87].

Most preclinical studies address CD36 indirectly, in the context of TSP binding. These studies exploit the antiangiogenic effect of TSP 1 and 2 via CD36 signalling, by using recombinant proteins, or TSR peptides, to compensate for loss of TSP in tumour cells. Controversies arose when migration and invasion of cancer cells seemed to be promoted although the primary tumour responded to treatment. A TSP-1 null/breast cancer mouse model demonstrated reduced pulmonary metastases, although there was no impact on primary tumour growth, indicative of effects on the metastatic [88]. The same group demonstrated that inclusion of the RFK sequence in the TSP recombinant protein impacts positively metastases reduction, but in relation to TGF*β* activation [88], and that, to some extent, loss of CD36 binding to TSP is compensated by the RFK sequence, in terms of antitumour effect. Another study on a mouse model of breast cancer, using a TSP-2-derived recombinant protein, reported both inhibition of primary tumour growth and reduction of lymph node and lung metastasis. Although the primary effect was positively correlated with CD36-induced mitochondrial apoptosis in endothelial cells and decreased neoangiogenesis; an antimetastatic effect was correlated with the RFK sequence and TGF*β* activation [89].

Results with another mouse model of metastatic breast cancer indicated that although CD36 expression in the whole primary tumour was downregulated, this alteration was related to loss of stromal receptor. This hypothesis was confirmed by normal expression of CD36 on isolated tumour cells [90].

In metastatic prostate cancer, CD36 was activated in tumour cells, which led to increased cell migration and invasion, linked to downstream activation of MAPK [91].

Ovarian tumour cells harvested from ascites of patients also express CD36, which was used by Wang et al. as a target for TSP-1-induced apoptosis and subsequent tumour shrinkage in a mouse xenograft model [92]. A recent study showed upregulation of CD36 in metastatic* versus* primary human ovarian tumours; moreover, blocking CD36 with monoclonal antibodies resulted in reduced tumour burden in a mouse xenograft model [83]. Furthermore, Russell et al. found that combined therapy with thrombospondin-1 type I repeats (3TSR) and chemotherapy induces regression and improves survival in a mouse model of ovarian cancer[93]. These results suggest that CD36 might offer an interesting therapy target, besides its putative biomarker role.

CD36 has been reported on glioblastoma cells as well, in a specific subset of stem-like cells, with role in stemness preservation and tumour initiation [85].

Recently, a study addressed directly the involvement of CD36 in tumour growth and metastasis, by overexpressing CD36 in oral squamous carcinoma cell lines. Tumour cells were then transplanted into immune-competent mice, showing significantly increased metastatic potential over their wild-type counterparts. Conversely, knocking CD36 down led to zero lymph node invasions. The same antimetastatic effect was obtained with CD36- targeting antibodies. While metastasis was prevented, or if already present, significantly reduced, primary lesions were not affected by the treatment [86]. Unlike previous reports, the work of Pascual et al. highlighted a possible cooperation between adipose tissue and tumour cells via CD36, which favours a predominantly lipidic metabolism. The link between increased lipidic profile and tumour progression was also highlighted in an obese mouse model of breast cancer [87]. The authors reported CD36 expression on some, but not all tumour cells, as well as downregulation of CD36 expression on endothelium of neovessels, presumably due to repressed CD36 gene transcription via PDK1-FOXO1 activation by lysophosphatidic acid.

The model that can be delineated so far is that a high CD36 level correlates with metastatic cancers and thus is poorly prognostic. However, blocking CD36 in a tumour system composed of tumour cells and stromal niche would equally affect both populations. An earlier work of deFillipis et al. [94] proposes that loss of CD36 in the pretumoural breast stroma creates a milieu favourable for tumour initiation or progression. Thus, targeting CD36 to prevent metastasis would have a protumorigenic impact on the surrounding stroma. Add to this the proangiogenic effect on the tumour itself, and the outcome will be, very likely, a thriving primary tumour that is possibly nonmetastatic.

**Figure 1:**
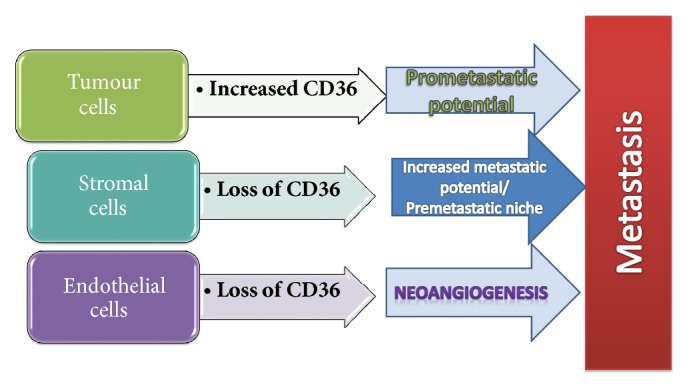
Involvement of CD36 in the metastatic process, related to the main three components of any tumour niche: the tumour cells, stromal cells, and vascularisation. CD36 expression in tumours prone to metastasis is different in each compartment, as demonstrated by animal models and pathology studies in patient samples.

These results highlight the importance of integration of models in a correct spatial context, in which stromal niches and tumour cells interact. From this perspective, which part is more important for metastasis? And, in consequence, which population of cells should be targeted in studies aiming at validation of CD36 as a prognostic biomarker (see Figure 1)? Which population holds greater prognostic value: CD36+ tumour cells, as proposed by Pascual et al., or CD36-depleted stroma, as proposed by deFillipis et al.?

Unfortunately, these aspects have not yet been covered by clinical studies, as discussed in the next section, although CD36 is emerging as a candidate prognostic biomarker in different types of epithelial cancers, alone or in panels with other proteins.

### 4.2. Human Studies

Data from animal models and* in vitro *human tumour cell lines point to CD36 as a metastasis-related indicator, prompting investigations on a larger scale in human tumour samples (Table 1). One of the first mentions of CD36 as a possible biomarker for cancer prognostics dates more than 15 years, when it was included in a panel of immunophenotyping for high risk for acute myeloblastic leukaemia [95].

In a study of inflammation and cancer, Rachidi et al. used a reductive approach by considering all epithelial cancers as oncoinflammatory events and looking for a common signature. Although CD36 did not meet the criteria for all seven types of cancers studied, a high CD36 gene expression level was proposed as a poor prognostic marker in colon and ovarian cancer when assessed in panel with other proteins [96].

Wang et al. found that 97% of ovarian cancers express CD36, as do 100% of lymph node metastases. Furthermore, the receptor's expression was increased with disease progression [92]. CD36 was also detected in liposarcoma and prostate and breast tumours; in the last type, translocation of the protein from the cytoplasm to cell membrane was related to oestrogen signalling [97].

Firm confirmation of the relationship between CD36 and metastasis came from a study of over 2500 cases of different types of cancers (a “pan-cancer” study) in which genes involved in metabolic rewiring towards aerobic glycolysis and* de novo *FA synthesis were assessed in metastatic tumours compared to primary tumours. The CD36 gene was frequently amplified in metastatic tumours and survival rates in the high-copy-number group were reduced when compared with low-copy-number patients [98].

## 5. Clinical Trials

Direct targeting of CD36 in tumour pathology has not yet been addressed in cancer-related clinical trials. Rather, its ability to bind TSP1 and modulate antiangiogenic responses was exploited therapeutically. Several clinical trials tested TSP-1 peptidomimetics specifically binding to CD36, but they were discontinued for lack of response or severe adverse effects [79]. Data gathered in preclinical models of various types of cancers taught that blocking CD36 might prove beneficial in stopping metastases from spreading. Lessons learned from other fields of successful clinical research show that humanized monoclonal antibodies are a valid option (reviewed in [99]). But a recent commentary estimates that development of antibodies against CD36, to be used in clinical trials, would take at least 4 years [100].

Continuing the pipeline of TSP peptidomimetics, in line with the lipid scavenger function of CD36, apolipoprotein A-I–mimetic peptides are being tested in preclinical trials, but mostly for noncancerous pathologies [102–104].

CD36 has been and continues to be investigated as a possible biomarker in metabolic diseases (obesity, insulin resistance, and diabetes type 2), cardiovascular diseases, and autoimmune/inflammatory conditions (Table 2).

The hope is that data gathered from these clinical trials will be highly informative about the pharmacological profile and side-effects of various types of CD-36 related compounds, for further repurposing in cancer therapy.

## 6. Future Perspectives in CD36-Related Tumour Biology

Plenty of data have been gathered to demonstrate CD36 involvement in metastasis spreading and, yet, novel and exciting avenues are still opening. Along with new reports of CD36 involvement in normal mitochondrial function [105], one could ask how CD36 increase impacts tumour cells energetic metabolism and the effect of CD 36 inhibition on bystander cells.

Further on, based on tumour animal models, the next step would be translation to human pathology. This process has already started, at least at the bioinformatics level, contributing to acknowledgment of CD36 as a possible prognostic biomarker for metastatic cancer, by compiling data from repositories and meta-analyses. So far, high levels of CD36 have been proposed as a poor prognostic marker for colon and ovarian cancer [96] as well as for breast cancer, lung small cell carcinoma, and urinary bladder carcinoma [86].

If the molecule is to be included in further clinical trials, validation in large cohorts remains to be accomplished, along with clarifications in some grey areas, such as site of detection of CD36 (stromal cells versus tumour cells) and proper quantification.

## Figures and Tables

**Table 1:** Involvement of CD36 in various types of cancer—data from human sample studies.

Type of cancer	Location within the tumour	Contribution of CD36	Refs
Breast cancer	Stroma tissue Decreased endothelial expression Tumour cells	Angiogenesis	[90] [87]
Prostate cancer	Tumour cells	Activation of MAPK signalling, pro-invasion	[91]
Ovarian cancer	Tumour cells harvested from patient ascites	Pro-metastatic	[92]
Colon cancer	Not specified	Decreased expression in metastatic cancer	[96]
Oral squamous cell carcinoma	Tumour cells	Favours lymph node and lung metastasis	[86]
Acute myeloid leukaemia	Tumour cells	Part of immunophenotyping panel used for patient stratification	[95]
Glioblastoma	Tumour cells	Maintenance of stemness features, tumour initiation	[85]
Hepatocellular carcinoma	Not mentioned	Increased CD36 is associated with epithelial-to-mesenchymal transduction	[101]

**Table 2:** Clinical trials investigating CD36 expression.

No	Conditions	Title	Interventions	Identifier
1	Healthy Young/Elderly	Effects of Native Whey or Milk Supplementation on Adaptations to 12 Weeks of Strength Training in Young and Elderly	Dietary Supplement: Native whey|Dietary Supplement: Milk|Other: Strength training	NCT03033953
		Anabolic Effects of Whey and Casein After Strength Training in Young and Elderly	Strength training|Dietary Supplement: Milk 1%|Dietary Supplement: Whey protein concentrate 80|Dietary Supplement: Native whey	NCT02968888

2	Taste Sensitivity|Fatty Acid Type	Fatty Acid Taste Thresholds: Caproic, Lauric, Oleic, Linoleic, Linolenic		NCT01996566

3	Obesity/Metabolic Syndrome	CD36 and Human Fat Taste Perception	No intervention	NCT02699567
		Metabolic and Cardiovascular Impact of CD36 Deficiency in African Americans		NCT02126735
		Study 8: Fat Perception in Humans (09-0873)		NCT01128400
		Study 19: Metabolically Normal and Metabolically Abnormal Obesity	Behavioral: overfeeding	NCT01184170

4	Insulin Resistance|Endothelial Dysfunction	CD36 in Nutrient Delivery and Its Dysfunction	Sildenafil Citrate (Viagra)	NCT03012386

5	Type 2 Diabetes or Obesity Without Diabetes	Search for Biological Markers of Orosensory Perception of Fatty Acids in Healthy Subjects and Possible Modifications in Patients With Type 2 Diabetes and in Obese Non-diabetic Patients.	Other: Measure the threshold of detection for linoleic acid|Other: Oral stimulation tests|Other: Venous blood samples|Other: Samples for genetic studies (ancillary study)	NCT02028975

6	Moderate Hypertriacylglycerolemic Subjects	Intervention With n3 LC-PUFA-supplemented Yogurt	Dietary Supplement: n3 long chain polyunsaturated fatty acids	NCT01244048

7	Diabetes Mellitus, Type 2|Dyslipidemia Associated With Type II Diabetes Mellitus|Percutaneous Coronary Intervention	Effects of Evolocumab on Platelet Reactivity in Patients With Diabetes Mellitus After Elective Percutaneous Coronary Intervention	Drug: Evolocumab|Drug: Placebo	NCT03258281

	Insulin Resistance|Diabetes|Cancer|Obesity|Inflammation	Study 14: Diet and Metabolic Inflammation	Other: Diet A|Other: Diet B	NCT02539355

	Renal Failure Chronic Requiring Hemodialysis|Metabolic Syndrome|Diabetes Mellitus Type 2|Hyperlipidemia	Study 16: Salusin-alpha - a New Factor in the Pathogenesis of Lipid Abnormalities in Hemodialysis Patients	Drug: Atorvastatin	NCT01448174

	Cardiovascular Abnormalities	Study 13: A Study of Immunological Biomarkers as Predictors of Cardiovascular Events	Other: blood tests	NCT02894060

	Coronary Artery Disease	Polymorphisms in CD36 and STAT3 Genes and Different Dietary Interventions Among Patients With Coronary Artery Disease	Dietary Supplement: Olive oil|Dietary Supplement: Nuts|Dietary Supplement: Control diet	NCT02202265

	Thrombocytopenia	Study 5: Identification of Donors of CD36-Deficient Platelets Among Japanese Individuals on the NIH Campus	None	NCT00015639

	Idiopathic Thrombocytopenic Purpura	The Effect of Eltrombopag on the Expression of Platelet Collagen Receptor GPVI in Pediatric ITP.	Drug: Eltrombopag|Drug: conventional	NCT03412188

	Myelodysplastic Syndromes	Study 22: Azacitidine and Erythropoietin Versus Azacitidine Alone for Patients With Low-Risk Myelodysplastic Syndromes	Azacitidine|Drug: Erythropoietin|Drug: Azacitidine (Monotherapy)	NCT00379912

	Rheumatoid Arthritis	Study 7: A New Mode of Action of Anti-TNF, Reverse Signaling, in Rheumatoid Arthritis	Diagnostic Test: Blood test	NCT03216928

	Rheumatoid Arthritis	Atherosclerosis in Rheumatoid Arthritis and Lupus: Restoring Cholesterol Balance		NCT01180361

	SLE|Rheumatoid Arthritis|Healthy Subjects	Study 15: Efferocytosis and Genomic Polymorphism in Autoimmune Diseases		NCT00364728

	HIV Infections	Study 12: Maraviroc 300 mg Twice Daily in HIV Negative Male Volunteers	Drug: Maraviroc	NCT00771823
